# Probabilistic Resumable Quantum Teleportation of a Two-Qubit Entangled State

**DOI:** 10.3390/e21040352

**Published:** 2019-04-01

**Authors:** Zhan-Yun Wang, Yi-Tao Gou, Jin-Xing Hou, Li-Ke Cao, Xiao-Hui Wang

**Affiliations:** 1 School of Electronic Engineering, Xi’an University of Posts and Telecommunications, Xi’an 710121, China; 2 School of Physics, Northwest University, Xi’an 710127, China; 3 Institute of Modern Physics, Northwest University, Xi’an 710127, China; 4 Shaanxi Key Laboratory for Theoretical Physics Frontiers, Xi’an 710127, China

**Keywords:** quantum teleportation, two-qubit entangled state, partially entangled state, local unitary operation, controlled-U gate

## Abstract

We explicitly present a generalized quantum teleportation of a two-qubit entangled state protocol, which uses two pairs of partially entangled particles as quantum channel. We verify that the optimal probability of successful teleportation is determined by the smallest superposition coefficient of these partially entangled particles. However, the two-qubit entangled state to be teleported will be destroyed if teleportation fails. To solve this problem, we show a more sophisticated probabilistic resumable quantum teleportation scheme of a two-qubit entangled state, where the state to be teleported can be recovered by the sender when teleportation fails. Thus the information of the unknown state is retained during the process. Accordingly, we can repeat the teleportion process as many times as one has available quantum channels. Therefore, the quantum channels with weak entanglement can also be used to teleport unknown two-qubit entangled states successfully with a high number of repetitions, and for channels with strong entanglement only a small number of repetitions are required to guarantee successful teleportation.

## 1. Introduction

Quantum teleportation (QT) is one of the most astonishing applications of quantum mechanics. This operable concept was originally presented by Bennett et al. in 1993 [1]. In this protocol, the sender (Alice) and the receiver (Bob) prearrange the sharing of an Einstein-Podolsky-Rosen (EPR) [2] correlated pair of particles. Alice makes a joint measurement on her EPR particle and the unknown quantum system; she then sends Bob the classical result of her measurement. Finally, Bob can convert the state of his EPR particle into an exact replica of the unknown state belonging to Alice by means of local operations and classical communication (LOCC). QT has been realized experimentally [3,4,5] and due to its fresh notion and latent applied prospects in the realm of quantum communication, various kinds of QT have been widely studied both theoretically [6,7,8,9] and experimentally [10,11,12].

From above researches, we can learn that a maximally entangled state as the quantum channel and two classical bits are the key ingredients for the deterministic teleportation with fidelity 1 [13,14]. However, in realistic situation, instead of the pure maximally entangled states, Alice and Bob usually share a mixed entangled state or a partially entangled state due to the decoherence. Teleportation using a mixed state as an entangled resource is, in general, equivalent to having a noisy quantum channel. As a mixed state can’t be purified to a Bell state [15,16,17], a quantum channel of mixed states could never provide a teleportation with fidelity 1 [18,19]. Therefore, only pure entangled pairs should be considered if we prefer an exact teleportation, even if it is probabilistic. Li et al. [20] put forward a partially entangled quantum channel to probabilistically teleport the quantum state of a single particle and extended this scheme to a multi-particle system. Much attention has been paid to this direction [21,22,23,24,25,26].

Recently, the field of entanglement has become an intense research area due to its key role in many applications of quantum information processing, such as precise measurement, quantum communication, quantum network and quantum repeater, etc. It is therefore an interesting question how we can teleport a pair of entangled particles. In 1999, Gorbachev et al. [27] proposed their protocol, teleportation of a two-qubit entangled state (TTES), by using a three-particle maximally entangled state of the type Greenberger-Horne-Zeilinger (GHZ). Two schemes for generalized TTES (GTTES) have been reported soon. In one of them, the protocol is assisted with a generalized three-particle entangled state as quantum channel, which is based on GHZ [28]. In the other probabilistic scheme, teleportation is completed with an entanglement swapping process [29], which is carried out via two pairs of partially entangled channels (TPEC). In their schemes, since the fidelity cannot reach 1, the maximal probabilities of exact teleportation was provided. According to the no-cloning theorem [30,31] and the irreversibility of quantum measurements [32,33], the information of the states to be teleported will be lost if the processes fail. Obviously, their destructive protocols do not offer the chance to repeat the process if TTES fails.

Roa et al. [34] presented a scheme for teleporting probabilistically an unknown pure state with optimal probability and without losing the information of the state to be teleported, and its advantage is that the unknown state is recovered by the sender when teleportation fails. This property offers the chance to repeat the teleportation process as many times as one has available quantum channels.

This paper is organized as follow. In Section 2, we present the probabilistic quantum teleportation of a two-qubit entangled state protocol, which uses two pairs of partially entangled particles as quantum channel. We verify that the optimal probability of successful teleportation is determined by the smallest superposition coefficient of these partially entangled particles. We introduce an optimal scheme, probabilistic resumable quantum teleportation of a two-qubit entangled state (RTTES) in Section 3, which is assisted with TPEC and has the advantage that the unknown entangled state can be recovered by the sender when the process fails. That is to say, if the sender and receiver have more than one partially entangled quantum channel, then the sender is able to teleport many times until RTTES is successful because the sender still have the unknown state undisturbed. In Section 4, we discuss the success probability of probabilistic TTES process. Finally, the conclusions are summarized in Section 5.

## 2. Probabilistic Teleportation of a Two-Qubit Entangled State

Suppose Alice has an arbitrary partially entangled pair, consisting of particles (1,2), which can be described as

(1) $${|\phi \rangle }_{12}=(x|00\rangle +{y|11\rangle )}_{12},$$

with ${\left|x\right|}^{2}+{\left|y\right|}^{2}=1$, where {|0〉,|1〉} are the eigenstates of the Pauli operator $\sigma_{z}=\left|0\rangle \langle 0\right|-\left|1\rangle \langle 1\right|$. Now Alice would like to teleport the unknown state ${|\phi \rangle }_{12}$ to Bob. She sets up two distant entangled pure states as quantum channel between herself (particles 3 and 5) and Bob (particles 4 and 6), which located in the following states, respectively:

(2) $$\left\{\begin{array}{ccc} {|\psi \rangle }_{34} & = & (a|00\rangle +{b|11\rangle )}_{34},\\ {|\psi \rangle }_{56} & = & (c|00\rangle +{d|11\rangle )}_{56}, \end{array}\right.$$

with |a|⩾|b|, ${\left|a\right|}^{2}+{\left|b\right|}^{2}=1$, |c|⩾|d|, ${\left|c\right|}^{2}+{\left|d\right|}^{2}=1$. Note that when |a|=|b| and |c|=|d|, the quantum channel is composed of two EPR pairs, and the deterministic teleportation can be achieved. This is the special case of our scheme. We demonstrate that, by using entanglement swapping [29], Alice can successfully transmit state ${|\phi \rangle }_{12}$ to Bob with certain probability.

The state of the whole system is given by

(3) $$\begin{array}{ccc} {|\Psi \rangle }_{123456} & = & {|\phi \rangle }_{12}⊗{|\psi \rangle }_{34}⊗{|\psi \rangle }_{56}\\ & = & \left[x(ac|000000\rangle +ad|000011\rangle +bc|001100\rangle +bd|001111\rangle \right)\\ & & +y(ac|110000\rangle +ad|110011\rangle +bc|111100\rangle +{bd|111111\rangle )]}_{123456}. \end{array}$$

Now Alice firstly performs local Bell-state measurements [35] on particles (1,3) and particles (2,5), respectively. The particles (4,6) belong to Bob will be projected to the corresponding quantum state, i.e., the above strategy is provided for the receiver to extract the quantum information by adopting a proper evolution. There are four outcomes in each Bell-state measurement, ($|\Phi^{\pm }\rangle =1/\sqrt{2}(|00\rangle \pm \left|11\rangle ),\right|\Psi^{\pm }\rangle =1/\sqrt{2}(|01\rangle \pm \left|10\rangle \right)$), so there are sixteen specific results in total.

For example, here we analyse the case that the results of measurement are $|\Phi^{+}\rangle_{13}$ and $|\Psi^{-}\rangle_{25}$, respectively. The particles (4,6) are collapsed into the following state

(4) $$\begin{array}{ccc} {|\Phi \rangle }_{46} & = & \langle \Phi_{13}^{+}|{\langle \Psi_{25}^{-}|\Psi \rangle }_{123456}\\ & = & \frac{1}{\sqrt{2}}\left(\langle 00\right|+{\langle 11|)}_{13}\frac{1}{\sqrt{2}}\left(\langle 01\right|-{\langle 10|)}_{25}{|\Psi \rangle }_{123456}\\ & = & \frac{1}{2}(xad|01\rangle -{ybc|10\rangle )}_{46}. \end{array}$$

Next, Alice informs Bob of the results by the classical communication and Bob performs a unitary operation $(|0\rangle \langle 0|+{|1\rangle \langle 1|)}_{4}⊗(|0\rangle \langle 1|-{|1\rangle \langle 0|)}_{6}$ on Equation (4). Then its state changes to

(5) $$\begin{array}{c} \frac{1}{2}(xad|00\rangle +{ybc|11\rangle )}_{46}. \end{array}$$

Without loss of generality, if the superposition coefficients satisfy |a|⩾|c|⩾|d|⩾|b|, we have |ac|⩾|bd|, |ad|⩾|bc|. In order to carry out the proper evolution, we need to introduce an auxiliary particle to Bob which initial state is ${|0\rangle }_{aux}$, and operate the following controlled unitary transformation under the basis ${\{|0\rangle }_{4}{|0\rangle }_{aux}{,|1\rangle }_{4}{|0\rangle }_{aux}{,|0\rangle }_{4}{|1\rangle }_{aux}{,|1\rangle }_{4}{|1\rangle }_{aux}\}$. The unitary transformation is described as the following controlled-U gate

(6) $$\begin{array}{c} U_{1}=\left|0\rangle \langle 0\right|⊗{\hat{U}}_{1}+\left|1\rangle \langle 1\right|⊗I, \end{array}$$

with ${\hat{U}}_{1}$ being a rotation in a π/2 angle around the ${\hat{n}}_{1}$ direction, specifically,

(7) $$\begin{array}{c} {\hat{U}}_{1}=e^{-i\frac{\pi }{2}}e^{i\frac{\pi }{2}{\hat{n}}_{1}\cdot \sigma },\;{\hat{n}}_{1}=\left(\sqrt{1-{\left(\frac{bc}{ad}\right)}^{2}},0,\frac{bc}{ad}\right), \end{array}$$

and $\sigma =(\sigma_{x},\sigma_{y},\sigma_{z})$. Note that the amplitudes a,b,c and *d* of the quantum channel have to be known in order to apply the above unitary transformation. The collective unitary transformation $U_{1}$ transforms the un-normalized state Equation (5) to the result

(8) $$\begin{array}{c} {|\Phi \rangle }_{46aux}=\frac{1}{2}bc(x|00\rangle +{y|11\rangle )}_{46}{|0\rangle }_{aux}+\frac{1}{2}ad\sqrt{1-{\left(\frac{bc}{ad}\right)}^{2}}{x|00\rangle }_{46}{|1\rangle }_{aux}, \end{array}$$

which is also un-normalized. Then we perform a measurement on the auxiliary particle. If the result of measurement is ${|1\rangle }_{aux}$, we can see that the teleportation fails with the state of qubits (4,6) transformed to the state $1/2ad\sqrt{{\left(1-bc/ad\right)}^{2}}x{|00\rangle }_{46}$ and no information regarding the initial state ${|\phi \rangle }_{12}$ is left. On the contrary, if the result of measurement is ${|0\rangle }_{aux}$, the state of particles (4,6) collapses to an exact replica of the teleported state ${|\phi \rangle }_{12}$. The teleportation is successfully accessed. The contribution of this un-normalized state can be expressed by the probabilistic amplitude of $(x|00\rangle +{y|11\rangle )}_{46}$ in Equation (8) as ${\left|(1/2)\times bc\right|}^{2}=\left(1/4\right)\times {\left|bc\right|}^{2}$.

Similarly, if Alice’s measurement results are $|\Psi^{+}\rangle_{13}$ and $|\Psi^{-}\rangle_{25}$, the particles (4, 6) are collapsed into the following state

(9) $$\begin{array}{ccc} {|\Phi \rangle }_{46} & = & \langle \Psi_{13}^{+}|{\langle \Psi_{25}^{-}|\Psi \rangle }_{123456}\\ & = & \frac{1}{2}(xbd|11\rangle -{yac|00\rangle )}_{46}. \end{array}$$

Bob operates a unitary operation $(|0\rangle \langle 1|+{|1\rangle \langle 0|)}_{4}⊗(|0\rangle \langle 1|-{|1\rangle \langle 0|)}_{6}$ on Equation (9) and changes it to

(10) $$\begin{array}{c} \frac{1}{2}(xbd|00\rangle +{yac|11\rangle )}_{46}. \end{array}$$

It should be noted that the unitary operation here is different from Equation (6):

(11) $$\begin{array}{c} U_{2}=\left|0\rangle \langle 0\right|⊗I+\left|1\rangle \langle 1\right|⊗{\hat{U}}_{2}, \end{array}$$

with ${\hat{U}}_{2}$ being a rotation in a π/2 angle around the ${\hat{n}}_{2}$ direction, specifically,

(12) $$\begin{array}{c} {\hat{U}}_{2}=e^{-i\frac{\pi }{2}}e^{i\frac{\pi }{2}{\hat{n}}_{2}\cdot \sigma },\;{\hat{n}}_{2}=\left(\sqrt{1-{\left(\frac{bd}{ac}\right)}^{2}},0,\frac{bd}{ac}\right). \end{array}$$

The state of particles (4,6) reduces to

(13) $$\begin{array}{c} {|\Phi \rangle }_{46aux}=\frac{1}{2}bd(x|00\rangle +{y|11\rangle )}_{46}{|0\rangle }_{aux}+\frac{1}{2}ac\sqrt{1-{\left(\frac{bd}{ac}\right)}^{2}}{y|11\rangle }_{46}{|1\rangle }_{aux}. \end{array}$$

**Figure 1:**
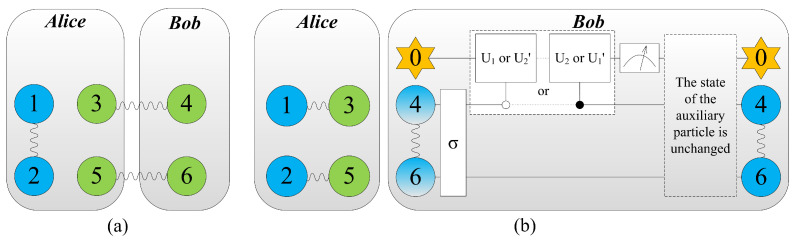
Colour online: blue represents the message to be teleported by Alice, and the information that needs to be extracted further is gradient. (**a**) System model for teleporting an arbitrary partially entangled pair consisting of particles (1, 2) by Alice. Particles (3, 4) and (5, 6) are partially entangled pairs. (**b**) By performing local Bell-state measurements on particles (1, 3) and (2, 5), the entanglement between particles (3, 4) and (5, 6) vanishes, while entanglement between particles (1, 3) and (2, 5) is built up. And then Bob extract the information from particles 4 and 6 via unitary operations and a von Neumann measurement. Eventually, the message has been teleported from Alice to Bob if and only if the state of auxiliary particle is unchanged after measurement.

So, for Equation (13), the probability of successful teleportation is $\left(1/4\right)\times {\left|bd\right|}^{2}$. Other measuring results can be discussed in the same way. The whole scheme is shown in Figure 1. We list all sixteen kinds of results, and show the corresponding operations respectively in Table 1, where the unitary operations $U_{1}^{\prime }=\left|0\rangle \langle 0\right|⊗I+\left|1\rangle \langle 1\right|⊗{\hat{U}}_{1}$ and $U_{2}^{\prime }=\left|0\rangle \langle 0\right|⊗{\hat{U}}_{2}+\left|1\rangle \langle 1\right|⊗I$. Synthesizing all cases, we obtain the total probability of successful teleportation being

(14) $$\begin{array}{c} P=\frac{1}{4}{\left|bc\right|}^{2}\times 8+\frac{1}{4}{\left|bd\right|}^{2}\times 8=2{\left|b\right|}^{2}. \end{array}$$

## 3. Resumable Quantum Teleportation of a Two-Qubit Entangled Sstate

Now we consider an improved project of GTTES to teleport a two-qubit entangled state via weak entanglement quantum channels. Firstly, in order to implement the protocol it will be required to apply a series of joint unitary transformations known as the controlled-NOT gate [36,37].

(15) $$\begin{array}{c} U_{NOT}^{\left(ij\right)}=|0_{i}\rangle \langle 0_{i}|⊗I_{j}+|1_{i}\rangle \langle 1_{i}|⊗\sigma_{x}^{\left(j\right)}, \end{array}$$

where $I_{j}$ is the identity operator of target system *j* and *i* is the control system. We apply the $U_{NOT}^{\left(31\right)}$ and $U_{NOT}^{\left(52\right)}$ controlled-NOT gates onto the system, so the state (3) becomes

(16) $$\begin{array}{ccc} {|\Gamma \rangle }_{123456} & = & U_{NOT}^{\left(31\right)}U_{NOT}^{\left(52\right)}{|\Psi \rangle }_{123456}\\ & = & \left[x(ac|000000\rangle +ad|010011\rangle +bc|101100\rangle +bd|111111\rangle \right)\\ & & +y(ac|110000\rangle +ad|100011\rangle +bc|011100\rangle +{bd|001111\rangle )]}_{123456}. \end{array}$$

Now we introduce two extra auxiliary qubits *m* and *n* set into the state ${|0\rangle }_{m}$ and ${|0\rangle }_{n}$ to Alice. So Alice can apply the $U_{NOT}^{\left(1m\right)}$ and $U_{NOT}^{\left(2n\right)}$ gates. The process of taking the state |Γ〉 to the |Υ〉 given by

(17) $$\begin{array}{ccc} {|\Upsilon \rangle }_{mn123456} & = & U_{NOT}^{\left(1m\right)}U_{NOT}^{\left(2n\right)}{|\Gamma \rangle }_{mn123456}\\ & = & \left[x(ac|00000000\rangle +ad|01010011\rangle +bc|10101100\rangle +bd|11111111\rangle \right)\\ & & +y(ac|11110000\rangle +ad|10100011\rangle +bc|01011100\rangle +{bd|00001111\rangle )]}_{mn123456}. \end{array}$$

To carry out our RTTES scheme, we shall apply the following controlled-U gate [38,39],

(18) $$\begin{array}{c} U^{\left(i,j\right)}=|00_{i}\rangle \langle 00_{i}|⊗U_{00}^{j}+|01_{i}\rangle \langle 01_{i}|⊗U_{01}^{j}+|10_{i}\rangle \langle 10_{i}|⊗U_{10}^{j}+|11_{i}\rangle \langle 11_{i}|⊗U_{11}^{j}, \end{array}$$

where the superscript *j* means the target system (particles 1 and 2) and the subscript *i* is the control system (particles 3 and 5). We define that the unitary matrices of $U_{00},U_{01},U_{10}$ and $U_{11}$ below:

(19) $$U_{00}=\left(\begin{array}{cccc} \frac{bd}{ac} & 0 & 0 & \sqrt{1-\frac{{\left(bd\right)}^{2}}{{\left(ac\right)}^{2}}}\\ 0 & 1 & 0 & 0\\ 0 & 0 & 1 & 0\\ \sqrt{1-\frac{{\left(bd\right)}^{2}}{{\left(ac\right)}^{2}}} & 0 & 0 & -\frac{bd}{ac} \end{array}\right),$$

(20) $$U_{01}=\left(\begin{array}{cccc} 1 & 0 & 0 & 0\\ 0 & \frac{bc}{ad} & \sqrt{1-\frac{{\left(bc\right)}^{2}}{{\left(ad\right)}^{2}}} & 0\\ 0 & \sqrt{1-\frac{{\left(bc\right)}^{2}}{{\left(ad\right)}^{2}}} & -\frac{bc}{ad} & 0\\ 0 & 0 & 0 & 1 \end{array}\right),$$

(21) $$U_{10}=I,$$

(22) $$U_{11}=I.$$

After applying the above $U^{\left(35,12\right)}$ gate, we can obtain the following state |Λ〉:

(23) $$\begin{array}{ccc} {|\Lambda \rangle }_{mn123456} & = & U^{\left(35,12\right)}{|\Upsilon \rangle }_{mn123456}\\ & = & {x|00\rangle }_{mn}(bd|00\rangle +\sqrt{{\left(ac\right)}^{2}-{\left(bd\right)}^{2}}{\left|11\rangle \right)}_{12}{|0000\rangle }_{3456}\\ & & +{y|11\rangle }_{mn}(-bd|11\rangle +\sqrt{{\left(ac\right)}^{2}-{\left(bd\right)}^{2}}{\left|00\rangle \right)}_{12}{|0000\rangle }_{3456}\\ & & +{x|01\rangle }_{mn}(bc|01\rangle +\sqrt{{\left(ad\right)}^{2}-{\left(bd\right)}^{2}}{\left|10\rangle \right)}_{12}{|0011\rangle }_{3456}\\ & & +{y|10\rangle }_{mn}(-bc|10\rangle +\sqrt{{\left(ad\right)}^{2}-{\left(bd\right)}^{2}}{\left|01\rangle \right)}_{12}{|0011\rangle }_{3456}\\ & & +{x|10\rangle }_{mn}{bc|10\rangle }_{12}{|1100\rangle }_{3456}\\ & & +{y|01\rangle }_{mn}{bc|01\rangle }_{12}{|1100\rangle }_{3456}\\ & & +{x|11\rangle }_{mn}{bd|11\rangle }_{12}{|1111\rangle }_{3456}\\ & & +{y|00\rangle }_{mn}{bd|00\rangle }_{12}{|1111\rangle }_{3456}. \end{array}$$

Next, we apply the $U_{NOT}^{\left(1m\right)}$ and $U_{NOT}^{\left(2n\right)}$ again, so the state (23) becomes

(24) $$\begin{array}{ccc} {|\Pi \rangle }_{mn123456} & = & U_{NOT}^{\left(1m\right)}U_{NOT}^{\left(2n\right)}{|\Lambda \rangle }_{mn123456}\\ & = & {|11\rangle }_{mn}{[|0000\rangle }_{3456}\sqrt{{\left(ac\right)}^{2}-{\left(bd\right)}^{2}}(x|11\rangle +{y|00\rangle )}_{12}\\ & & +{|0011\rangle }_{3456}\sqrt{{\left(ad\right)}^{2}-{\left(bc\right)}^{2}}(x|10\rangle +{y|01\rangle )}_{12}]\\ & & +{|00\rangle }_{mn}{[bdx|00\rangle }_{12}{|0000\rangle }_{3456}+{bcx|01\rangle }_{12}{|0011\rangle }_{3456}\\ & & +{bcx|10\rangle }_{12}{|1100\rangle }_{3456}+{bdx|11\rangle }_{12}{|1111\rangle }_{3456}\\ & & -{bdy|11\rangle }_{12}{|0000\rangle }_{3456}-{bcy|10\rangle }_{12}{|0011\rangle }_{3456}\\ & & +{bcy|01\rangle }_{12}{|1100\rangle }_{3456}+{bdy|00\rangle }_{12}{|1111\rangle }_{3456}]. \end{array}$$

In the following, we will analyse the different measurement results of the above final state.

If the qubits (m, n) are projected to ${|11\rangle }_{mn}$, after Alice performs a Bell-state measurement on her joint system consisting of particles (3, 5), she can recover the teleported entangled particle pair ${|\phi \rangle }_{12}$ of qubits (1, 2) by means of local operations. The detailed situations are summarized in Table 2, i.e., for the outcome ${|11\rangle }_{mn}$, the teleportation fails but the process performs the projection ${|\phi \rangle }_{12}\to {|\phi \rangle }_{12}$ on itself.

On the contrary, if the qubits (m,n) are projected to ${|00\rangle }_{mn}$, we can write out the system of residual particles as

(25) $$\begin{array}{ccc} {|\Pi \rangle }_{123456} & = & {bdx|00\rangle }_{12}{|0000\rangle }_{3456}+{bcx|01\rangle }_{12}{|0011\rangle }_{3456}\\ & & +{bcx|10\rangle }_{12}{|1100\rangle }_{3456}+{bdx|11\rangle }_{12}{|1111\rangle }_{3456}\\ & & -{bdy|11\rangle }_{12}{|0000\rangle }_{3456}-{bcy|10\rangle }_{12}{|0011\rangle }_{3456}\\ & & +{bcy|01\rangle }_{12}{|1100\rangle }_{3456}+{bdy|00\rangle }_{12}{|1111\rangle }_{3456}. \end{array}$$

Then, we apply the controlled-NOT gates $U_{NOT}^{\left(31\right)}$ and $U_{NOT}^{\left(52\right)}$ on the system consisting of the residual particles, and the state |Π〉 becomes

(26) $$\begin{array}{ccc} {|\Omega \rangle }_{123456} & = & U_{NOT}^{\left(31\right)}U_{NOT}^{\left(52\right)}{|\Pi \rangle }_{123456}\\ & = & {bdx|00\rangle }_{12}{|0000\rangle }_{3456}+{bcx|00\rangle }_{12}{|0011\rangle }_{3456}\\ & & +{bcx|00\rangle }_{12}{|1100\rangle }_{3456}+{bdx|00\rangle }_{12}{|1111\rangle }_{3456}\\ & & -{bdy|11\rangle }_{12}{|0000\rangle }_{3456}-{bcy|11\rangle }_{12}{|0011\rangle }_{3456}\\ & & +{bcy|11\rangle }_{12}{|1100\rangle }_{3456}+{bdy|11\rangle }_{12}{|1111\rangle }_{3456}. \end{array}$$

Then Alice carry out the joint Bell-state measurements on the systems consisting of particles (1, 3) and (2, 5). For instance, here we assume that the results of the measurements are $|\Phi^{+}\rangle_{13}$ and $|\Psi^{-}\rangle_{25}$. The particles (4,6) will be projected to

(27) $$\begin{array}{ccc} {|\Phi \rangle }_{46} & = & \langle \Phi_{13}^{+}|{\langle \Psi_{25}^{-}|\Omega \rangle }_{123456}\\ & = & \frac{1}{\sqrt{2}}\left(\langle 00\right|+{\langle 11|)}_{13}\frac{1}{\sqrt{2}}\left(\langle 01\right|-{\langle 10|)}_{25}{|\Omega \rangle }_{123456}\\ & = & \frac{1}{2}bc(x|01\rangle -{y|10\rangle )}_{46}. \end{array}$$

**Figure 2:**
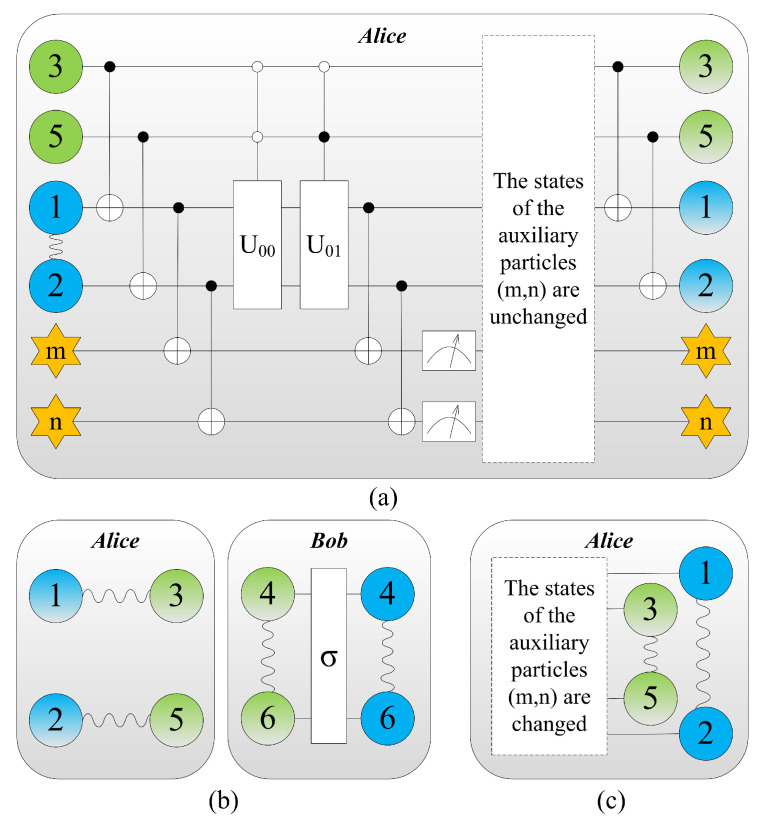
Colour online: blue represents the message to be teleported by Alice, and the information that needs to be extracted further is gradient. The initial state of the system is the same as that of (a) in Figure 1. (**a**) Alice introduces two extra auxiliary qubits *m* and *n* with the states ${|0\rangle }_{m}$ and ${|0\rangle }_{n}$ respectively to extract information stored in qubits 1 and 2. A series of joint unitary transformations known as the controlled-NOT gate and the controlled-U gate are performed by Alice. (**b**) Alice carry out the joint Bell-state measurements on the systems consisting of particles (1,3) and (2,5). The entanglement between particles (3, 4) and (5, 6) vanishes, while entanglement between particles (1, 3) and (2, 5) is built up. And then Bob extract the information from particles 4 and 6 via unitary operations and a von Neumann measurement. Eventually, the message has been teleported from Alice to Bob if and only if the states of auxiliary particles are unchanged after measurement. (**c**) The states of the auxiliary particles (m,n) are changed, the teleportation fails. Alice performs a Bell-state measurement on her joint system consisting of particles (3,5), she can recover the teleported entangled particle pair ${|\phi \rangle }_{12}$ of qubits (1, 2) by means of local operations.

We can see that the projection of qubits (m, n) to ${|00\rangle }_{mn}$ allows one to achieve the RTTES process by means of LOCC. We list all 16 kinds of results and the corresponding operations in Table 3. The whole scheme is shown in Figure 2.

## 4. Discussion

We learn that the teleported two-qubit entangled state ${|\phi \rangle }_{12}$ can be recovered if RTTES process fails. Thus we can repeat a single RTTES process as many times as one has available quantum channels. And if $a=b=1/\sqrt{2}$ and $c=d=1/\sqrt{2}$ in Equation (2), the quantum channel reduces to maximally entangled states and hence the total probability equals 1 [27]. On the other hand, it is obvious that the RTTES is successful with probability $2{\left|b\right|}^{2}$, which is the same as Equation (14) in Section 2. Therefore, our RTTES scheme does not increase the probability of success by a single experiment, but provides a chance to repeat the RTTES process many times until this process is successful. It can be regarded as a Bernoulli experiment. After realizing *N* tries, the probability of having *k* successful events is in the form of binomial distribution PN,k=Nk[2b2]k[1−2b2]N−k. Thus, we obtain the total probability of success as follows

(28) $$\begin{array}{c} P=\sum_{k=1}^{N}P_{N,k}=1-{\left[1-2b^{2}\right]}^{N}. \end{array}$$

**Figure 3:**
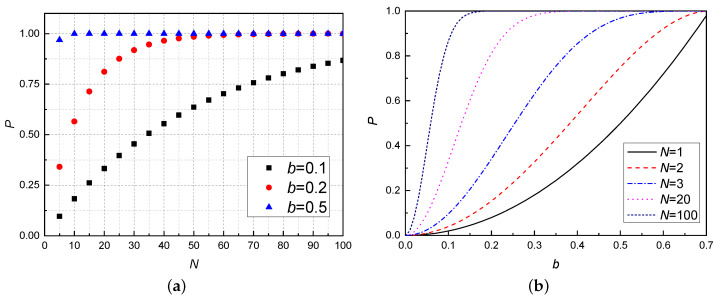
Colour online: total success probability of RTTES, as a function of *b* and *N*, here *N* means we performed RTTES *N* tries. (**a**) Total success probability of RTTES, as a function of the *N* for different values of *b*. The larger the coefficient *b*, the stronger the entanglement of quantum channel and the greater the probability of successful teleportation. (**b**) Total success probability of RTTES, as a function of *b* for different values of *N*. The probability increases significantly for N higher than 1. We can draw the conclusion that the quantum channels with weak entanglement (*b* = 0.1 or 0.2) can also be used to teleport successfully with a high number of repetitions, and for channels with strong entanglement (*b* = 0.5) only a small number of repetitions are required to guarantee successful teleportation.

The coefficient *b* in Equation (2) represents the degree of entanglement of the quantum channel. When the quantum channel is a maximum entanglement state, i.e., $a=b=1/\sqrt{2}$, $c=d=1/\sqrt{2}$, see Figure 3b, the RTTES process becomes deterministic, which is consistent with the result in Ref. [29]. Note that the success probability of probabilistic RTTES process will increase significantly for many tries. These results are summarized in Figure 3.

## 5. Conclusions

In summary, we have proposed two teleportation schemes, the generalized probabilistic teleportation of a two-qubit entangled state (GTTES) and the probabilistic resumable teleportation of a two-qubit entangled state (RTTES), which use partially entangled pairs as quantum channel. In the standard deterministic protocol, a maximally entangled quantum channel is necessary for the success of teleportation. In real world, however, it is well known that the coupling between quantum systems and surrounding environment is inevitable [40], e.g., different kinds of decoherence, dephasing, and dissipation mechanisms reduce purity and entanglement of the channel. Therefore, sender and receiver may not shared a maximally entangled state but a partially entangled state. For this reason, GTTES is more general and practical. The differences between GTTES and TTES are that GTTES introduces an auxiliary particle, and need to perform local unitary operations before Bell-state measurements. We show that the optimal success probability of GTTES is only dependent on the smallest superposition coefficient of the partially entangled quantum channels. In other words, the success probability of GTTES cannot reach to 1. If GTTES fails, the state to be teleported will be destroyed. In addition, taking into account that an unknown state cannot be cloned, the above GTTES protocol do not offer the chance to repeat the process if GTTES fails. An improved scheme of GTTES (RTTES) is proposed. The advantage of this approach is that we are able to try repeatedly until the RTTES is successful. It is conformed to Bernoulli experiment, and total success probability of teleportation increases significantly by attempting many times. Finally, weak entanglement can be used to teleport a two-qubit entangled state effectively via RTTES. Our research also provides insights into the role of entanglement in quantum teleportation that it can be regarded as a key resource.

## Figures and Tables

**Table 1:** The specific situation of the measurement results and the corresponding unitary operations in GTTES.

Alice’s Measurement Results	Probability	Bob’s Unitary Operation
$|\Phi^{+}\rangle_{13}{|\Phi^{\pm }\rangle }_{25}$	$\frac{1}{4}{\left|bd\right|}^{2}$	$[I_{4}⊗(|0\rangle \langle 0|\pm {|1\rangle \langle 1|)}_{6}]\cdot U_{2}^{\prime }$
$|\Phi^{-}\rangle_{13}{|\Phi^{\pm }\rangle }_{25}$	$\frac{1}{4}{\left|bd\right|}^{2}$	$[I_{4}⊗(|0\rangle \langle 0|\mp {|1\rangle \langle 1|)}_{6}]\cdot U_{2}^{\prime }$
$|\Phi^{+}\rangle_{13}{|\Psi^{\pm }\rangle }_{25}$	$\frac{1}{4}{\left|bc\right|}^{2}$	$[I_{4}⊗(|0\rangle \langle 1|\pm {|1\rangle \langle 0|)}_{6}]\cdot U_{1}$
$|\Phi^{-}\rangle_{13}{|\Psi^{\pm }\rangle }_{25}$	$\frac{1}{4}{\left|bc\right|}^{2}$	$[I_{4}⊗(|0\rangle \langle 1|\mp {|1\rangle \langle 0|)}_{6}]\cdot U_{1}$
$|\Psi^{+}\rangle_{13}{|\Phi^{\pm }\rangle }_{25}$	$\frac{1}{4}{\left|bc\right|}^{2}$	$\left[(|0\rangle \langle 1\right|\pm {|1\rangle \langle 0|)}_{4}⊗I_{6}]\cdot U_{1}^{\prime }$
$|\Psi^{-}\rangle_{13}{|\Phi^{\pm }\rangle }_{25}$	$\frac{1}{4}{\left|bc\right|}^{2}$	$\left[(|0\rangle \langle 1\right|\mp {|1\rangle \langle 0|)}_{4}⊗I_{6}]\cdot U_{1}^{\prime }$
$|\Psi^{+}\rangle_{13}{|\Psi^{\pm }\rangle }_{25}$	$\frac{1}{4}{\left|bd\right|}^{2}$	$\left[(|0\rangle \langle 1\right|+{|1\rangle \langle 0|)}_{4}⊗(|0\rangle \langle 1|\pm {|1\rangle \langle 0|)}_{6}]\cdot U_{2}$
$|\Psi^{-}\rangle_{13}{|\Psi^{\pm }\rangle }_{25}$	$\frac{1}{4}{\left|bd\right|}^{2}$	$\left[(|0\rangle \langle 1\right|+{|1\rangle \langle 0|)}_{4}⊗(|0\rangle \langle 1|\mp {|1\rangle \langle 0|)}_{6}]\cdot U_{2}$

**Table 2:** The specific outcomes of measurement and the corresponding unitary operations.

Alice’ Result	Probability	Operation
${|00\rangle }_{35}$	${|\left(ac\right)}^{2})-{\left(bd\right)}^{2}|$	$(|0\rangle \langle 1|+{|1\rangle \langle 0|)}_{1}⊗(|0\rangle \langle 1|+{|1\rangle \langle 0|)}_{2}$
${|01\rangle }_{35}$	${|\left(ad\right)}^{2}-{\left(bc\right)}^{2}|$	$(|0\rangle \langle 1|+{|1\rangle \langle 0|)}_{1}⊗(|0\rangle \langle 0|+{|1\rangle \langle 1|)}_{2}$

**Table 3:** The specific situation of the measurement results and the corresponding unitary operations in RTTES.

Alice’s Measurement Results	the State of Particles (4,6)	Bob’s Unitary Operation
$|\Phi^{+}\rangle_{13}{|\Phi^{\pm }\rangle }_{25}$	$\frac{1}{2}bd(x|00\rangle \pm {y|11\rangle )}_{46}$	$I_{4}⊗(|0\rangle \langle 0|\pm {|1\rangle \langle 1|)}_{6}$
$|\Phi^{+}\rangle_{13}{|\Psi^{\pm }\rangle }_{25}$	$\frac{1}{2}bc(x|01\rangle \pm {y|10\rangle )}_{46}$	$I_{4}⊗(|0\rangle \langle 1|\pm {|1\rangle \langle 0|)}_{6}$
$|\Phi^{-}\rangle_{13}{|\Phi^{\pm }\rangle }_{25}$	$\frac{1}{2}bd(x|00\rangle \mp {y|11\rangle )}_{46}$	$I_{4}⊗(|0\rangle \langle 0|\mp {|1\rangle \langle 1|)}_{6}$
$|\Phi^{-}\rangle_{13}{|\Psi^{\pm }\rangle }_{25}$	$\frac{1}{2}bc(x|01\rangle \mp {y|10\rangle )}_{46}$	$I_{4}⊗(|0\rangle \langle 1|\mp {|1\rangle \langle 0|)}_{6}$
$|\Psi^{+}\rangle_{13}{|\Phi^{\pm }\rangle }_{25}$	$\frac{1}{2}bc(x|10\rangle \mp {y|01\rangle )}_{46}$	$(|0\rangle \langle 1|\mp {|1\rangle \langle 0|)}_{4}⊗I_{6}$
$|\Psi^{-}\rangle_{13}{|\Phi^{\pm }\rangle }_{25}$	$\frac{1}{2}bc(x|10\rangle \pm {y|01\rangle )}_{46}$	$(|0\rangle \langle 1|\pm {|1\rangle \langle 0|)}_{4}⊗I_{6}$
$|\Psi^{+}\rangle_{13}{|\Psi^{\pm }\rangle }_{25}$	$\frac{1}{2}bd(x|11\rangle \mp {y|00\rangle )}_{46}$	$(|0\rangle \langle 1|\mp {|1\rangle \langle 0|)}_{4}⊗(|0\rangle \langle 1|+{|1\rangle \langle 0|)}_{6}$
$|\Psi^{-}\rangle_{13}{|\Psi^{\pm }\rangle }_{25}$	$\frac{1}{2}bd(x|11\rangle \pm {y|00\rangle )}_{46}$	$(|0\rangle \langle 1|\pm {|1\rangle \langle 0|)}_{4}⊗(|0\rangle \langle 1|+{|1\rangle \langle 0|)}_{6}$
